# Adverse childhood experiences and hormonal contraception: Interactive impact on sexual reward function

**DOI:** 10.1371/journal.pone.0279764

**Published:** 2023-01-17

**Authors:** Andrew M. Novick, Joel Stoddard, Rachel L. Johnson, Korrina A. Duffy, Lily Berkowitz, Vincent D. Costa, Mary D. Sammel, C. Neill Epperson

**Affiliations:** 1 Department of Psychiatry, School of Medicine, University of Colorado, Aurora, Colorado, United States of America; 2 Department of Biostatistics and Informatics, Colorado School of Public Health, University of Colorado, Aurora, Colorado, United States of America; 3 Department of Behavioral Neuroscience, Oregon Health and Science University, Portland, Oregon, United States of America; 4 Department of Family Medicine, School of Medicine, University of Colorado, Aurora, Colorado, United States of America; PLOS, UNITED KINGDOM

## Abstract

The current literature suggests that some women are uniquely vulnerable to negative effects of hormonal contraception (HC) on affective processes. However, little data exists as to which factors contribute to such vulnerability. The present study evaluated the impact of prepubertal adverse childhood experiences (ACEs) on reward processing in women taking HC (N = 541) compared to naturally cycling women (N = 488). Participants completed an online survey assessing current and past HC use and exposure to 10 different adverse childhood experiences (ACEs) before puberty (ACE Questionnaire), with participants categorized into groups of low (0–1) versus high (≥2) prepubertal ACE exposure. Participants then completed a reward task rating their expected and experienced valence for images that were either erotic, pleasant (non-erotic), or neutral. Significant interactions emerged between prepubertal ACE exposure and HC use on expected (*p* = 0.028) and experienced (*p* = 0.025) valence ratings of erotic images but not pleasant or neutral images. Importantly, follow-up analyses considering whether women experienced HC-induced decreases in sexual desire informed the significant interaction for expected valence ratings of erotic images. For current HC users, prepubertal ACEs interacted with HC-induced decreased sexual desire (*p* = 0.008), such that high ACE women reporting decreased sexual desire on HC showed substantially decreased ratings for anticipated erotic images compared to both high prepubertal ACE women without decreased sexual desire (*p* < 0.001) and low prepubertal ACE women also reporting decreased sexual desire (*p* = 0.010). The interaction was not significant in naturally cycling women reporting previous HC use, suggesting that current HC use could be impacting anticipatory reward processing of sexual stimuli among certain women (e.g., high prepubertal ACE women reporting HC-induced decreases in sexual desire). The study provides rationale for future randomized, controlled trials to account for prepubertal ACE exposure to promote contraceptive selection informed by behavioral evidence.

## Introduction

Approximately 400 million people around the world use hormonal contraception (HC) [1]. Yet, it remains unknown exactly how these agents influence the brain and affective function as findings in the literature often conflict. For example, whereas a recent large meta-analysis of nearly 10,000 participants revealed that those taking HC, regardless of formulation, experience decreased sexual desire compared to those not taking HC [2], another review suggested that negative effects on sexual desire are less common, with the majority of women experiencing no effect or even improvements in sexual desire [3]. These discordant findings could be reconciled if data showed that a subset of women are particularly vulnerable to HC’s influence on affective functions underlying sexual desire. Recently, we reported that high levels of adverse childhood experiences (ACEs) were associated with a greater risk of discontinuing HC use due to decreased sexual desire, suggesting a possible relationship between ACEs and HC-induced deficits in reward processing for sexual stimuli [4]. The present study evaluates whether ACEs and HC use have additive or interactive effects with one another in influencing reward processing (as measured by a behavioral task).

Several lines of evidence suggest that ACEs are associated with changes in reward processing. The majority of research in both humans and animals demonstrates that early life stress, especially when such stressors are experienced prior to puberty, result in decreased anticipation and motivation for various types of rewards as well as decreased hedonic experience of reward (for review see [5]). This may be due to the fact that prior to puberty, the rapid development of subcortical regions governing the anticipation and experience of reward (such as the nucleus accumbens) may be more vulnerable to stress-induced changes [6,7]. Our group has previously found that the behavioral and neural consequences of ACEs are particularly apparent when gonadal hormones are in a state of flux [8–10]. Specifically, high ACE women are more vulnerable to premenstrual syndrome [11], premenstrual dysphoric disorder [12], perinatal depression [13], and mood and cognitive changes during menopause (12–15) [8–10]. These findings echo several studies in animals that deficits in reward processing only occur with the combination of early life stress and a subsequent stressor or pharmacological challenge, positing an interactive model [14–16]. Thus, while women with ACEs might be more vulnerable to deficits in reward processing at baseline, it is also possible that they may only occur in the context of hormonal changes that influence the brain, such as taking HC.

Some evidence suggests that HC on its own impacts processing of sexual rewards–although no studies have tested whether the effects are accentuated among or even limited to women with a high ACE history. In animal models, the common oral contraceptive agent levonorgestrel with ethinyl estradiol decreases social interaction and copulatory behavior [17]. In human studies, women using HC experience less enjoyment of erotic images [18] and rate their partner’s attractiveness as lower [19]. These behavioral findings are supported by neuroimaging evidence of HC-induced blunting of reward-related regions, such as the insula when anticipating erotic stimuli [20] and the nucleus accumbens when rating the attractiveness of their partner [19]. Yet, the effect of HC on reward may be specific to sexual stimuli. During anticipation of reward in the monetary incentive delay task (a nonsocial, nonsexual task), HC use actually increased activation of the insula [21] in contrast to its effect on insula activation when anticipating erotic stimuli [20].

While endogenous estradiol and progesterone play a role in facilitating reward processing, their synthetic analogues used in HC possess distinct pharmacology that could negatively impact reward function. Unlike endogenous progesterone, most progestins in HC are not metabolized to allopregnanolone [22,23]–a neurosteroid that can facilitate reward behavior by enhancing dopamine release in reward-sensitive regions, such as the nucleus accumbens [24]. Additionally, ethinyl estradiol, the estrogen used in most oral contraceptive agents, decreases the availability of androgens relative to endogenous estradiol [25], which would be expected to reduce androgen facilitation of reward neurobiology [26]. Whether the pharmacological consequences of HC on reward function are more pronounced in or limited to women with a history of high ACEs, however, remains an open question.

The present study sought to investigate the effects of HC and ACEs on reward processing of two types of affectively positive stimuli–erotic and pleasant stimuli–with the goal of elucidating how these factors impact reward processing for sexual and nonsexual stimuli and, by extension, how this might affect vulnerability to the negative behavioral side effects of HC. We tested this by recruiting a large sample of women from the general population who were either taking HC or not. We then measured their expectations and experience of three types of images (erotic, pleasant, and neutral) using an online reward task. We hypothesized that ACEs experienced prior to puberty would be associated with HC-related deficits in reward behavior related to erotic stimuli.

## Methods

### Participants

We recruited a sample of reproductive-aged (18–40 years) cisgender women (assigned female at birth with female gender identification) using listservs, social media, and ResearchMatch, a national registry of volunteers who consent to be contacted by researchers regarding studies for which they may be eligible. To be eligible for the study, women had to self-report being in good health, without severe or unstable physical, neurological, or psychiatric conditions (women reporting non-severe or stable medical and psychiatric conditions were eligible with the exception of those reporting bipolar disorder or schizophrenia); those who reported current use of HC (HC group) had to have been using the same HC for the past three months; and those who reported no current use of HC (No HC group) had to have been free of HC use for the past three months. Thus, the No HC group consisted of women who were former users of HC as well as those who had never used HC. Those in the No HC group were required to have a regular menstrual cycle between 21 and 35 days based on self-report. Additional information on inclusion/exclusion criteria can be found in the S1 File. The surveys and reward task were conducted remotely online via the REDCap platform hosted by the University of Colorado–Anschutz Medical Campus. Participants were enrolled in a lottery with an 8.3% chance of winning $100. All participants provided consent to participate via an electronic consent form. The Colorado Multiple Institutional Review Board reviewed and approved this study (protocol #20–1583).

### Self-report measures

Participants completed surveys on demographic information and health history, primarily to determine eligibility. Questions about current and past HC use included (if applicable): the specific type of HC they use(d), whether they experience(d) certain side effects, and reasons why they discontinued past HC use. Participants currently taking HC were asked to indicate what side effects they experienced, with one of the options being “decreased libido/sexual desire” due to their HC. If participants had previously taken HC, they were asked what the reason for discontinuation was, with one of the options listed as “decreased libido/sexual desire.”

Participants also completed the Adverse Childhood Experience Questionnaire (ACE-Q) [27]. The ACE-Q asks participants to indicate which of 10 types of adversity occurred before the age of 18. The ACE-Q includes items on abuse (emotional, physical, and sexual), neglect (emotional and physical), and household/family dysfunction (parental separation or divorce, household domestic violence, household substance abuse, parental mental illness, and member of household imprisoned). For each endorsed item, participants were asked their age at onset of the adverse experience. All experiences occurring 2 years or more prior to their age at first menstruation were considered prepubertal ACEs [8]. For each participant, prepubertal ACE score was calculated by adding the number of endorsed experiences that occurred prior to puberty. Previously, our group has found differences in the impact of prepubertal and postpubertal ACEs on risk for depression during the menopause transition [8]. We specifically chose to focus on prepubertal ACEs given evidence in humans and animals that stressors experienced prior to puberty result in decrements in reward processing while those experienced during or after puberty are more likely to have no effect or even enhance reward seeking behavior (for review see [5]). Given that the negative impact of ACEs are not typically apparent at the group-level unless participants have 2 or more ACEs [4,8–10,28,29], we dichotomized participants into a low ACE group (0 or 1) and a high ACE group (2 or more) in line with previous studies [4,8–10,28,29]. Further rationale for this approach (versus treating ACEs as a continuous variable) can be found in the S1 File.

### Reward task

**Fig 1:**
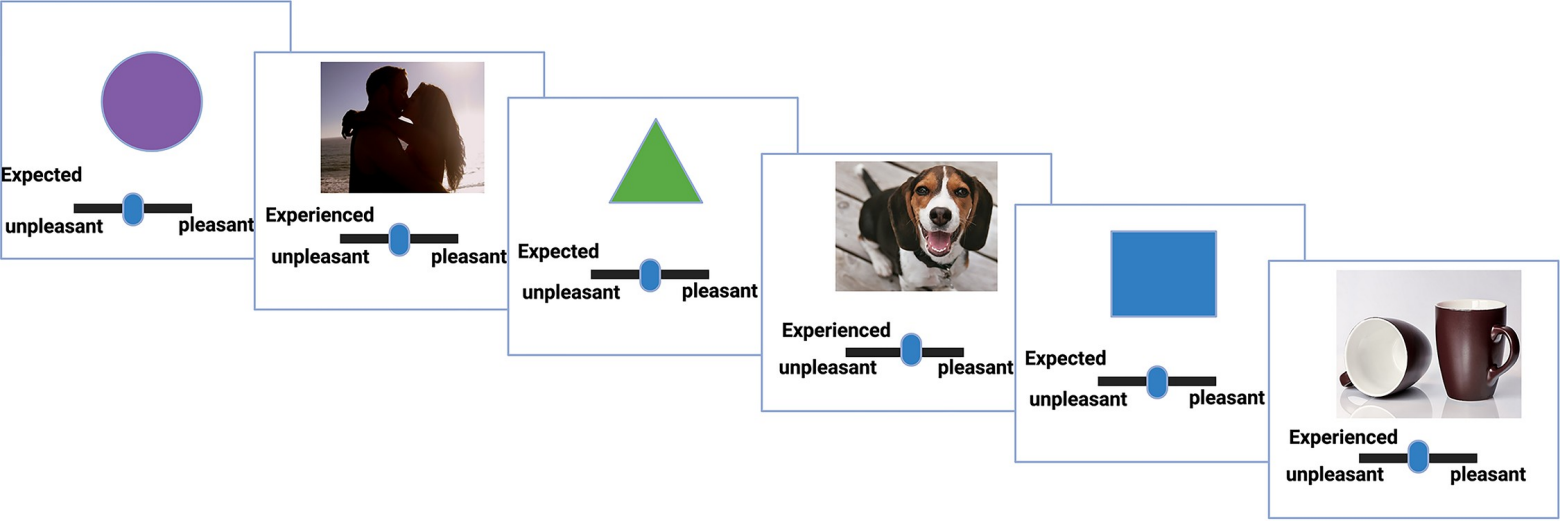
Task overview demonstrating cues and example images for erotic, pleasant, and neutral categories.

Participants were required to rate their expectation and experience of reward for three types of images (erotic, pleasant, and neutral) in a reward task adapted from Jepma et al [30] and Willroth et al [31]. Expectation and experience of rewarding stimuli are considered important yet distinct aspects of reward processing that map onto desire/motivation and hedonic pleasure, respectively [5,32]. Three distinct geometric shapes acted as cues that indicated the category of image that the participant would subsequently view (purple circle = erotic; green triangle = pleasant; blue square = neutral). On presentation of the cue, the subject was asked to rate how pleasant they expected the upcoming image to be using a sliding scale ranging from 0 = most unpleasant to 100 = most pleasant–a rating that we refer to as the “expected valence.” After this, they were presented with the image and were asked to rate how pleasant the image actually was using the same sliding scale–a rating that we refer to as the “experienced valence” (Fig 1). Participants were instructed that “pleasant” in this context represented things that they like, enjoy, or think are positive. Participants rated their expected and experienced valence of 60 unique images: 20 erotic images, 20 pleasant images (e.g., nature scenes, cute animals), and 20 neutral images (e.g., forks, furniture). The same images were presented to each participant, but participants were randomly assigned to one of four randomization orders. Prior to starting the task, participants were given instructions that included three practice cue/image pairs (one from each category) to introduce them to the task and the relationship between each cue and image category. REDCap was used to present the images and capture participant input. The task was not timed. When the participant completed an expected/experienced rating, the task would advance to present the image or cue. All images were selected from publicly available images on the internet or from affective picture sets currently used in research [33–35]. All images used were validated for appropriate valence in a separate group of 130 reproductive-aged females (details on validation can be found in S1 File). Similar types of images (and their cues) have been found to activate regions of the brain associated with reward [20,36,37].

### Statistical analysis

Demographics were summarized with means and standard deviations for continuous variables and with frequencies and percentages for categorical variables within the entire analysis cohort and stratified by HC group and ACE group. Differences between groups were tested with two-sample t-tests with unequal variances for continuous variables and with Fisher’s exact tests for categorical variables due to small sample sizes in some cells.

Valence ratings (expected or experienced) were modeled for each image type (erotic, pleasant, neutral) in linear mixed-effects models using the nlme package in R 3.6.3 [38,39]. All models included an interaction between HC and ACE groups as fixed effects and a random intercept for each participant. For models with significant interactions at *α* = 0.05, post-hoc pairwise comparisons were estimated. Reported p-values were not adjusted for multiple comparisons.

To determine relevant covariates, we evaluated which demographic variables were associated with expected and experienced image ratings at a *p* < 0.10 and which variables differed between HC and ACE groups (*p* < 0.10). Variables that differed according to HC use (Table 1) and/or ACE group (S1 Table in S1 File) and were associated with image ratings (S2 Table in S1 File) were subsequently included in our main analyses. Covariates included age, race, sexual orientation, and education; image randomization group was also included in all adjusted models to control for any potential order effects of image presentation (S3 Table in S1 File).

**Table 1:** Demographics of study completers stratified by whether they use hormonal contraception (HC).

Demographics N (%) or mean (SD)	Study completers (N = 1029)	No HC (N = 488)	HC (N = 541)	p-value
Age	28.7 (5.2)	29.0 (5.6)	28.5 (4.8)	0.125
Race				0.014
Asian	78 (7.6%)	46 (9.4%)	32 (5.9%)	
Black or African American	40 (3.9%)	26 (5.3%)	14 (2.6%)	
White	844 (82.0%)	383 (78.5%)	461 (85.2%)	
Other/multiracial	67 (6.5%)	33 (6.8%)	34 (6.3%)	
Ethnicity				0.416
Hispanic or Latino	74 (7.2%)	39 (8.0%)	35 (6.5%)	
Non-Hispanic or Non-Latino	938 (91.2%)	439 (90.0%)	499 (92.2%)	
Other or multiple ethnicities	17 (1.7%)	10 (2.0%)	7 (1.3%)	
Sexual orientation				0.091
Heterosexual	802 (77.9%)	373 (76.4%)	429 (79.3%)	
Bisexual	156 (15.2%)	75 (15.4%)	81 (15.0%)	
Homosexual/gay/lesbian	35 (3.4%)	24 (4.9%)	11 (2.0%)	
Other	36 (3.5%)	16 (3.3%)	20 (3.7%)	
Relationship status				0.001
In a relationship	684 (66.5%)	300 (61.5%)	384 (71.0%)	
Single	345 (33.5%)	188 (38.5%)	157 (29.0%)	
Household income				0.727
Less than $25,000	76 (7.4%)	40 (8.2%)	36 (6.7%)	
$25,000 - $75,000	410 (39.8%)	197 (40.4%)	213 (39.4%)	
$75,000 - $200,000	461 (44.8%)	214 (43.9%)	247 (45.7%)	
$200,000 or more	82 (8.0%)	37 (7.6%)	45 (8.3%)	
Work status				0.312
Essential	320 (31.1%)	144 (29.5%)	176 (32.5%)	
Not essential	709 (68.9%)	344 (70.5%)	365 (67.5%)	
Highest education level				<0.001
High school diploma or less	172 (16.7%)	107 (21.9%)	65 (12.0%)	
College degree	499 (48.5%)	224 (45.9%)	275 (50.8%)	
Master’s/professional degree	358 (34.8%)	157 (32.2%)	201 (37.2%)	
Prepubertal ACE	1.2 (1.6)	1.2 (1.7)	1.1 (1.5)	0.070
Low (0–1)	743 (72.2%)	340 (69.7%)	403 (74.5%)	0.094
High (2+)	286 (27.8%)	148 (30.3%)	138 (25.5%)	

Continuous variables are summarized with means (standard deviations), with differences among groups tested using two-sample t-tests. Categorical variables are summarized with frequencies (percentages), with differences among groups tested using Fisher’s exact tests.

Exploratory analyses were conducted using the same framework to test whether there were interactions between ACE group and HC-induced decreases in sexual desire on reward behavior to erotic images. Analyses were conducted separately for current HC users, who were asked about current HC side effects, and former HC users, who were asked about side effects that contributed to HC discontinuation.

For sample size and power considerations, we relied on preliminary data collected during validation of our affective images (see S1 File for full description of our image validation procedure). For the erotic images selected for this study, the average standard deviation of valence ratings by 34 reproductive-aged women was 1.2. Given that the study population for the current study was intended to be more diverse, a pooled standard deviation of 2.0 for mean valence ratings of erotic images was assumed for these calculations. Assuming 80% power and 2-sided type 1 error of 5% and assuming a mean difference in valence of 0.5 between women taking HC and those not taking HC, we would need approximately 200 subjects in each group. Given that we anticipated that women would be on various types of HC, we inflated our proposed sample size to 500 women on HC and 500 women not on HC to take this variance into account. PASS 2020 software version 20.0.5 was used for power calculations.

## Results

### Sample characteristics

#### Characteristics of hormonal contraception users and non-users

Of the 1029 women who completed the study, women were approximately evenly split between those currently (HC: N = 541) and not currently (No HC: N = 488) using HC. The women in the sample largely identified as white (82%), and white women were more likely to be currently using HC than women of other races (*p* = 0.014). The No HC group endorsed an overall lower level of education, with 22% reporting a high school diploma or less versus 12% in the HC group. Randomization to the four image order groups for the reward task did not differ between HC users and No HC users (*p* = 0.878). For more details, see Table 1.

#### Characteristics of women with a low versus high number of prepubertal adverse childhood experiences

Of the 1809 total ACEs reported across all participants, 1188 (65.7%) occurred prior to puberty. Over a quarter of the sample (27.8%) reported 2 or more ACEs occurring prior to puberty and were thus characterized as being in the high ACE group. The distribution of low and high ACE women in the HC and No HC groups was not statistically different (*p* = 0.094, Table 1). There was a trend towards a higher average number of ACEs in the No HC group (1.2) versus HC group (1.1) (*p* = 0.070, Table 1). Women in the low and high ACE groups differed in ethnicity (*p* = 0.001; S1 Table in S1 File), education (*p* = 0.001; S1 Table in S1 File), and sexual orientation (*p =* 0.002; S1 Table in S1 File). With regards to sexual orientation, the high ACE group versus low ACE group had a higher percentage of individuals identifying as bisexual (22.4% vs. 12.4%) as well as individuals identifying as homosexual/gay/lesbian (3.8% vs. 3.2%). Assignment of image randomization group for the reward task did not differ between ACE groups (*p* = 0.137). For more details, see S1 Table in S1 File.

### Consideration of hormonal contraception type

Oral contraceptive pills (OCPs) and hormonal intrauterine devices (IUDs) were the most commonly reported types of HC used, reported by 45.9% and 42.4% of the HC group, respectively. To test whether it was appropriate to group all HC users together regardless of HC type, we first grouped HC users into three categories: hormonal IUDs (46%), progestin + estrogen hormonal contraceptives (e.g., combined OCPs, Xulane patch, Nuvaring; 43%), and progestin only hormonal contraceptives (e.g., progestin only OCPs, Nexplanon implant, medroxyprogesterone injections; 10%). Without adjusting for any potential covariates, we then tested the interaction of HC type by ACE group on expected and experienced valence ratings of erotic, pleasant, and neutral images. No significant interaction emerged for either expected valence ratings (neutral *p* = 0.162; pleasant *p =* 0.521; erotic *p* = 0.171) or experienced valence ratings (neutral *p* = 0.379; pleasant *p* = 0.138; erotic *p* = 0.619). In a subsequent analysis, HC type also did not differ by ACE group (*p* = 0.966; see Table S1 in S1 File). Therefore, we combined HC types together.

#### Influence of prepubertal adverse childhood experiences (ACE) group and hormonal contraception (HC) use on expected and experienced image ratings

**Fig 2:**
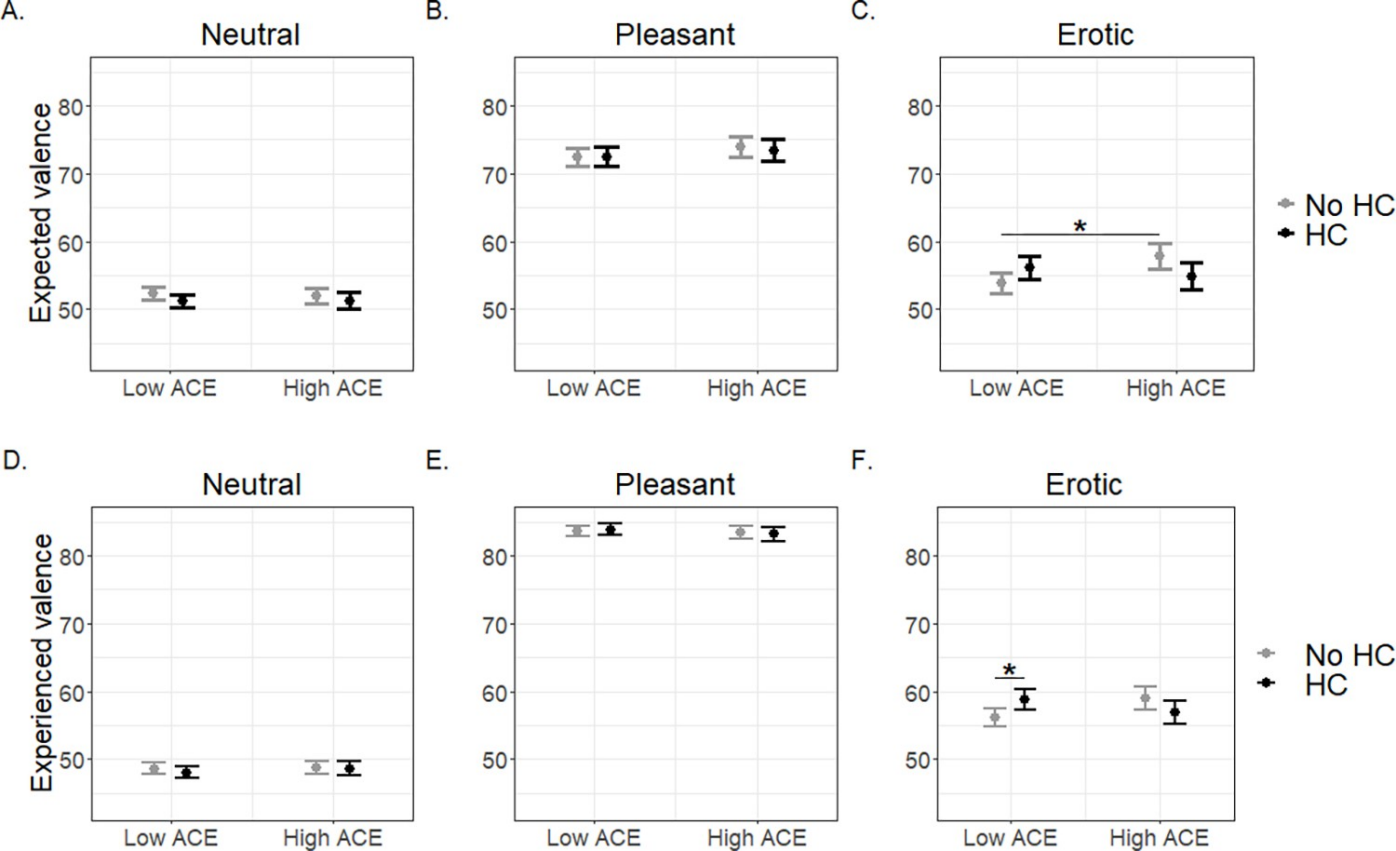
A-F. Expected and experienced valence ratings for different image types according to hormonal contraception (HC) use and prepubertal adverse childhood experience (ACE) group. A-C: For erotic images, but not neutral and pleasant images, hormonal contraception (HC) use and prepubertal ACEs interacted to predict expected valence (*p* = 0.028). Specifically, in the group not currently using HC, those in the high ACE group anticipated a higher valence for erotic images than those in the low ACE group (*p* = 0.020). D-F: For erotic images, but not neutral and pleasant images, hormonal contraception (HC) use and prepubertal ACEs interacted to predict experienced valence (*p* = 0.025). Specifically, in the low prepubertal ACE group, those currently using HC experienced a higher valence for erotic images than those not currently using HC (*p* = 0.020). Low ACE = 0 to 1 ACEs. High ACE = 2+ ACEs.

Significant interactions of ACE group (low vs. high) by HC use (HC vs. No HC) emerged for both expected (*p* = 0.028; Table 2; Fig 2C) and experienced (*p* = 0.025; Table 2; Fig 2F) valence ratings of erotic images. For expected valence of erotic images, in the No HC group, women in the high versus low ACE group had significantly higher expected valence (*p* = 0.020; Fig 2C). For experienced valence of erotic images, among women in the low ACE group, those taking HC rated their experience of viewing erotic images as significantly more positive than those in the No HC group (*p* = 0.020; Fig 2F). For neutral and pleasant images, no significant ACE by HC interactions were found for either expected or experienced valence ratings (*p*s > 0.05; Table 2). All models adjusted for randomization group, age, race, sexual orientation, and education (see Statistical Analysis in the Methods for a discussion on covariate selection). Effects of all covariates for this analysis are described in S4 Table in S1 File.

**Table 2:** Hormonal contraception (HC) use interacted with prepubertal adverse childhood experiences (ACEs) to predict expected and experienced valence of erotic images but not neutral or pleasant images.

				Low ACE				High ACE		
Condition Type	Interaction p-value	Interaction *χ*^2^, *df*	HC estimate (95% CI)		Z	p-value	HC estimate		Z	p-value
Expected Neutral	0.802	0.06, 1	-1.12 (-2.64, 0.40)		-1.44	0.149	-0.75 (-3.19, 1.68)		-0.61	0.543
Expected Pleasant	0.792	0.07, 1	0.02 (-2.08, 2.12)		0.02	0.983	-0.50 (-3.86, 2.85)		-0.30	0.768
Expected Erotic	**0.028**	4.86, 1	2.28 (-0.22, 4.78)		1.79	0.074	-2.98 (-6.98, 1.02)		-1.46	0.144
Experienced Neutral	0.715	0.13, 1	-0.52 (-1.83, 0.80)		-0.77	0.440	-0.06 (-2.16, 2.04)		-0.05	0.957
Experienced Pleasant	0.713	0.14, 1	0.26 (-1.10, 1.62)		0.37	0.710	-0.22 (-2.39, 1.95)		-0.20	0.843
Experienced Erotic	**0.025**	**5.04, 1**	**2.63** **(0.41, 4.85)**		**2.33**	**0.020**	-2.13 (-5.67, 1.42)		-1.18	0.240

The interaction effect was tested separately for each condition type, and all models controlled for randomization group, race, age, education, and sexual orientation. The interaction of HC use and prepubertal ACE group was significant for erotic images but not neutral or pleasant images. Low ACE = 0 to 1 ACEs. High ACE = 2+ ACEs.

#### Influence of prepubertal adverse childhood experiences (ACE) group and self-reported decreased sexual desire related to hormonal contraception (HC)-use on expected and experienced erotic image ratings

Given the significant ACE group (low vs. high) by HC use (No HC vs. HC) interaction on erotic image ratings, we next tested how ACE status might differentially affect reward behavior with erotic images depending on whether women were currently experiencing or had experienced HC-induced decreases in sexual desire. Because the questions addressing HC-induced decreases in sexual desire queried current experience in HC users and reasons for discontinuation in women with past HC use, we conducted separate analyses for each of these subgroups to test the interaction of ACE group and HC-related decreased sexual desire on expected and experienced valence of erotic images.

**Fig 3:**
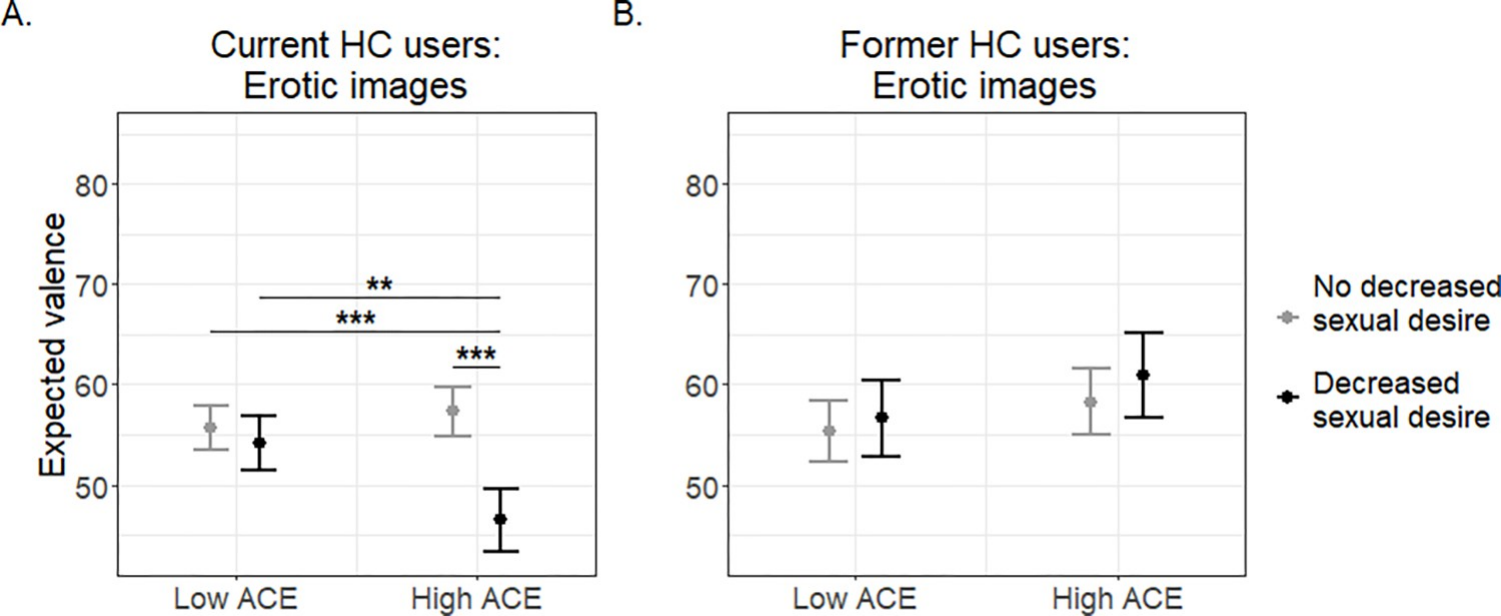
Interaction of prepubertal adverse childhood experiences (ACEs) group and self-reported decrease in sexual desire attributed to hormonal contraception (HC) use on expected valence ratings of erotic images. A: Current HC users who either endorse a decrease in sexual desire related to HC use or do not. B: Former HC users who either endorse having stopped using HC due to a decrease in sexual desire or discontinued due to other reasons. Only women currently using HC showed a significant interaction of ACE group by HC-related decreased sexual desire on expected valence ratings of erotic images (*p* = 0.008*)*. This effect is driven by the fact that high ACE women who report experiencing decreased sexual desire on HC show a substantially lower anticipated valence of erotic images than the other three groups of current HC users (** *p* = 0.010, *** p < 0.001).

For women currently taking HC, a significant interaction emerged for expected (*p* = 0.008; S5 Table in S1 File; Fig 3) but not experienced (*p* = 0.195, S5 Table in S1 File) valence ratings of erotic images. Pairwise comparisons revealed that, within the high ACE group, those who reported decreased sexual desire on HC had significantly lower expected ratings compared to those who did not report decreased sexual desire on HC (*p* < 0.001; S5 Table in S1 File; Fig 3). Those in the high ACE group who reported decreased sexual desire also demonstrated significantly lower expected ratings for erotic images compared to those in the low ACE group with (*p =* 0.010; Fig 3) and without (*p* < 0.001; Fig 3) decreased sexual desire. Expected valence ratings of erotic images did not differ between women in the low ACE group based on their reported effects of HC on their sexual desire (*p* = 0.429, S5 Table in S1 File, Fig 3). All models adjusted for randomization group, age, race, sexual orientation, and education (see Statistical Analysis in the Methods for a discussion on covariate selection). Effects of all covariates for this analysis are described in S6 Table in S1 File.

For women in the high ACE group currently taking HC, no differences in type of HC used (*p =* 0.202, S8 Table in S1 File) or demographic variables (S9 Table in S1 File) emerged between those who did and did not report decreased sexual desire on their current HC.

For women in the No HC group who were former HC users, there were no significant interactions between ACE group and self-reported discontinuation of HC due to decreased sexual desire on either expected (*p* = 0.761; S5 Table in S1 File; Fig 3) or experienced (*p* = 0.335; S5 Table in S1 File) valence ratings of erotic images. Effects of all covariates for this analysis are described in S7 Table in S1 File.

## Discussion

The results of the present study provide the first evidence that HC differentially impacts sexual reward processing in an ACE-dependent manner. Specifically, prepubertal ACE history interacted with HC use to influence anticipation and experience of erotic stimuli, but we found no evidence of this with stimuli that were merely pleasant. Most notably, women in the high ACE group who endorsed decreased sexual desire while currently using HC were less likely to report anticipated enjoyment of erotic images compared to the other three groups of current HC users (high ACE with no decreased sexual desire, low ACE with decreased sexual desire, and low ACE with no decreased sexual desire). These results add to recent work from our group demonstrating that ACEs are associated with HC discontinuation due to decreased sexual desire [4] and suggest that HC-induced changes in reward function related to sexual stimuli may underlie this increased discontinuation risk among high ACE women. Identifying a subgroup of women who demonstrate greater vulnerability to the effects of HC on affective function like processing of sexual rewards helps clarify some of the inconsistent findings in the literature on the effect of HC on sexual desire [2,3]. In addition, our study motivates research that could assist with a more personalized approach for prescribing in which a patient’s individual factors, such as ACEs, could be taken into account to help predict side effects.

As hypothesized, prepubertal ACEs significantly moderated the effect of HC use on expected and experienced ratings for erotic images, but we found no evidence of this for neutral or pleasant images. Work in both animals and humans suggests that stressors experienced prior to sexual maturity are associated with deficits in reward expectation and responsiveness while those occurring during or after puberty may have no effect or even enhance sensitivity to reward [5]. Thus, it was novel to find that among women not currently taking HC, the high prepubertal ACE group showed a higher average expected valence rating for erotic images compared to the low ACE group. Importantly, previous human research on reward processing and ACEs has focused on non-sexual stimuli [5,40], and given additional brain regions and circuits involved in sexual reward [41], the present result poses less of a contradiction to available literature. Furthermore, higher erotic image valence ratings in high ACE versus low ACE women not taking HC were only when anticipating and not when viewing erotic images. Expectation and experience are two related, but distinct, components of reward processing that map onto separate neurobiological functions and affective constructs [5,32,42]. While expectation of reward tends to relate to desire, anticipation, and motivation and is associated with dopamine function, experienced reward is associated with consummatory pleasure and the opioid system [43]. One possibility for our finding is that prepubertal ACEs might sensitize naturally cycling women’s anticipatory and motivation processes related to sexual stimuli, without influencing processes related to actual reward responsiveness for sexual stimuli, emphasizing the importance of considering expected versus experienced reward in future studies when trying to elucidate the biological basis of reward processing and the impact of childhood sexual trauma or other types of adversity. Importantly, some evidence shows that victimization in childhood is associated with increased risky sexual behaviors [44] and that childhood sexual abuse can be associated with later sexual preoccupation [45]. However, conflicting evidence suggests that childhood sexual abuse can also be associated with sexual aversion [45,46]. In our study, we did not focus solely on childhood sexual abuse in our characterization of high and low ACE groups (although see S10 and S11 Tables for supplemental analyses on sexual abuse) and it remains unclear whether the increased expected valence ratings for erotic images in high ACE women not taking HC relate to sexual behaviors and attitudes (in either positive or negative ways). To gain insight into this finding, future studies examining childhood adversity and sexuality should include measures of sexual behavior and attitudes along with assessments of sexual reward processing, while considering HC use as a potential moderator.

Among low prepubertal ACE women, those taking HC had higher average experienced valence ratings for erotic images compared to those not taking HC, suggesting greater responsiveness to erotic stimuli without differences in anticipatory processes. While this finding emphasizes the differential effects of HC according to prepubertal ACE status, it potentially conflicts with small studies not accounting for ACE history that found that, compared to naturally cycling women, women using oral contraception spent less time looking at images of genitals [47], rated them as less sexually appealing [18], and rated their own male partners as less attractive (which was associated with decreased activation in reward-related brain regions) [19]. These differential findings may be due in part to the type of erotic images used in the present study, which included neither pictures of genitalia nor the participant’s actual partner (see S1 File for details about erotic images used). Additional potential reasons for discrepancies between our findings and findings in previous studies could be that we included all possible types of HC and accounted for ACE history as well. The literature suggests that, for oral HC in particular, antiandrogenic effects [3,48,49] as well as decreases in allopregnanolone [17] may interfere with sexual reward function. Although the pharmacologic effects of some forms of HC may hinder sexual reward function, the literature suggests numerous psychological benefits of HC that could actually improve sexual function–such as decreased anxiety over unplanned pregnancy, reduced pain during intercourse, and improved complexion [3]. In addition, women who use HC may differ from women who do not use HC in ways that contribute to greater enjoyment of erotic stimuli. For example, one study reported that users of HC demonstrated a less restrictive sexual morality and more interest in erotic images compared to non-users [50]. Our study is the first to unpack the relationship between HC use and enjoyment of erotic stimuli when accounting for history of childhood adversity and suggests the importance of considering this variable in studies of HC and sexual stimuli.

A notable finding was that women in the high prepubertal ACE group taking HC who endorsed HC-induced decreases in sexual desire showed lower anticipatory reward for erotic images compared to the three other groups: high ACE women who did not endorse decreased sexual desire on HC, low ACE women who endorsed decreased sexual desire on HC, and low ACE women who did not endorse decreased sexual desire (S5 Table in S1 File, Fig 3). Importantly, low ACE women on HC who reported HC-induced decreases in sexual desire did not show a reduction in anticipated reward from erotic images compared with low ACE women on HC without self-reported decreases in sexual desire. Therefore, lower anticipatory reward function in women experiencing HC-induced decreases in sexual desire appear to be ACE-dependent. Also, former HC users who had discontinued HC due to decreased sexual desire did not demonstrate lower anticipated enjoyment of erotic images compared to those who had discontinued for other reasons regardless of ACE group. Thus, even though our study was observational rather than a randomized clinical trial, our findings suggest that HC could be driving differences in anticipatory reward for sexual stimuli among high ACE women who report sexual side effects from their current HC product. Future longitudinal work, ideally with neurobiological measures, is needed to confirm whether HC discontinuation results in normalization of reward dysfunction in susceptible populations. If confirmed, HC use in the context of prepubertal adversity might be an example of the “two-hit hypothesis” in which changes in brain function and behavior following early life adversity only emerge after a subsequent “stressor” (whether the “stressor” is a drug or psychosocial exposure) [51].

The mechanism by which prepubertal ACEs influence reward expectation is likely related to the early maturation of brain regions governing reward, such as the nucleus accumbens, making such regions vulnerable to stress-induced alterations early in development [6,7]. This could be further compounded by HC-induced changes in hormones. HC influences testosterone, estradiol, and allopregnanolone–all of which impact reward behavior and have receptors within the nucleus accumbens [24,26,52]. Potential evidence for the role of HC-induced hormonal changes in driving problems with sexual function comes from a study showing that women experiencing decreases in sexual desire and arousal on one particular HC formulation had improved sexual function when they switched to another formulation that has less influence on androgen levels [49]. Nevertheless, the overall association between HC-induced changes in androgens and sexual desire is typically weak [3,48], suggesting that perhaps only a subgroup of women may have sexual functioning that is sensitive to HC-induced decreases in testosterone. Future research should test whether HC-induced decreases in testosterone are more likely to interfere with sexual functioning in women reporting substantial childhood adversity, particularly with onset in the prepubertal window.

Besides decreases in androgens, another possible mechanism for how HC could affect reward function is through their effects on progesterone. Unlike endogenous progesterone, the progestin component of most HCs are not metabolized to allopregnanolone, which binds to GABA receptors to enhance mood, decrease anxiety, and facilitate reward [24,53]. Despite the fact that animal studies have demonstrated that HC-induced decreases in allopregnanolone are associated with increased anxiety [54] and decreased sexual motivation [17], one prospective HC study in humans did not find associations between decreased allopregnanolone and mood changes [55]. However, this study did not assess sexual desire, reward function, or ACE history, which is a critical limitation given that ACEs may be an important moderator of certain reward functions when taking HC and women with a high ACE history may be more sensitive to decreases in allopregnanolone. For example, women with a high ACE history are more susceptible to depression in the postpartum period–a time when allopregnanolone declines dramatically [13,56].

### Strengths and limitations

One of the strengths of the present study includes its use of a reward task that differentiates anticipatory reward function from reward responsiveness across various types of stimuli. Another strength is its large sample, allowing us to test the effect of HC use on reward function by comparing current HC users reporting HC-induced decreases in sexual desire with former HC users who discontinued HC due to decreased sexual desire. The differential effects on reward anticipation to erotic stimuli found in current users versus former users provide strong rationale for a randomized clinical trial investigating the impact of HC use on reward function in women with differing exposure to prepubertal ACE who report HC-induced decreases in sexual desire.

The cross-sectional nature of the present study limits the extent to which conclusions can be drawn regarding causal effects of HC on reward processing, but our provocative findings motivate the need for further research, including randomized controlled trials. Although we tested for ACE group by HC use interactions on different image categories separately and only found effects for erotic images, due to power considerations, we did not test a full model that included stimuli type in the interaction term. As such, we cannot say definitively that our results are specific to erotic stimuli.

Our study was administered completely over the internet, which enabled us to have a large sample but also led to a rather racially and ethnically homogenous sample. Another downside of online studies is that participants may be less honest and engaged. To address this possibility, we included quality control measures in our survey to guard against the use of bots and mindless selection of responses. Even with honest and engaged participants, retrospective reporting of ACEs and reasons for HC discontinuation has its limitations, and thus future studies should utilize prospective measures if possible. While the way that we defined prepubertal ACEs (as those occurring two years prior to menstruation) has previously been used to demonstrate the importance of ACE timing [8], using retrospective self-report is less precise than prospective hormonal measurements or physical examination to confirm onset of puberty. Also, because participants answered questions on HC side effects (such as decreased libido) prior to the reward task, demand characteristics might have influenced their responses to erotic stimuli. Future studies could investigate whether ACEs impact susceptibility to demand characteristics in general and thus address whether demand characteristics are relevant to the present studies and others like it.

The remote nature of the present study, while allowing for large numbers of participants, limited our ability to conduct hormonal measurements that would have enabled us to better account for the effects that variation in endogenous hormones, such as estradiol, can have on reward function [57]. Grouping all types of HC together in the absence of hormonal measurements limited our ability to generate more precise hypotheses on potential biological mechanisms since different types of HC differ in their systemic exposure as well as their pharmacologic effects. Previous studies on HC and reward have mainly focused on oral HC [18,19,21,21,47], which might be more potent at influencing sexual reward processes compared to HC formulations with less systemic effects such as IUDs [3,4]. Although we did not find a significant effect of HC type on image ratings, our study likely did not have the power to differentiate between types of HC, especially because, even within the same type of HC (e.g., pill, IUD), various formulations were included. Menstrual cycle phase (corresponding to different levels of estradiol and progesterone) in non-users of HC can impact reward sensitivity [58,59], and although we collected data on time since last menstrual period, we did not find an association between menstrual cycle phase and expected or experienced valence ratings of erotic images (S12 Table in S1 File). Precise and reliable determination of menstrual cycle phase usually combines self-report with physiological measures (i.e., body temperature, hormone testing) [60], which was not possible in this remote, internet-based study. Objective determination of menstrual cycle phase and other hormonal measurements (such as testosterone, estradiol, and allopregnanolone) in future studies will be important to determine potential biological mechanisms for our effects and to account for possible sources of variance.

In our analyses, p-values for statistical tests were not adjusted for multiple comparisons for two main reasons. First, our primary research question focused on the impact of ACEs and HC use on reward behavior related to erotic stimuli. The neutral and pleasant stimuli were included as a reference. Therefore, adjusting for multiple comparisons would have diminished our capacity to detect significant effects for our outcome of interest. Second, a strong positive correlation of expected and experienced valance was anticipated and was confirmed in our analyses. As such, adjusting for multiple outcomes risks over adjusting because our outcomes were correlated. Therefore, the statistical significance should be balanced against the magnitude of our reported effects [61].

Finally, while we adjusted for variables (such as age, race, education, and sexual orientation) that differed between groups and were associated with valence ratings of images, other potential confounders that were not assessed likely existed—perhaps related to characteristics that differ between those who stay on HC, those who stop using it, and those who never try it. In addition, there may have been additional characteristics that differed between HC users and non-users depending on ACE history that could have influenced the results. Given the potential for confounding, prospective, randomized clinical trials will be a critical next step.

### Conclusions and future directions

The results of the present study demonstrate for the first time that prepubertal ACEs influence the effects of HC on reward anticipation and response to erotic stimuli. Specifically, current HC use is associated with decrements in expected enjoyment of erotic stimuli only in women in the high ACE group who reported HC-induced decreases in sexual desire. Current HC users in the low ACE group who likewise reported HC-induced decreases in sexual desire did not show this effect, suggesting that HC may be detrimental to processes related to anticipated sexual rewards in the context of a higher ACE load, which may contribute to discontinuation among these women. By identifying differential effects of HC on sexual reward behavior based on ACE history, these results help reconcile the inconsistent literature regarding the impact of HC on sexual side effects and reward processing. Our results identify a subgroup of women who may be particularly vulnerable to reduced sexual reward function while using HC, which serves as a critical first step towards inspiring future investigations on how childhood adversity may shape a woman’s experience if she chooses to use HC.

## Supporting information

S1 File Contains additional information regarding inclusion/exclusion criteria, survey questions, selection and validation of images used in the reward task, and selection of covariates. (DOCX)
