# Supplementary material for: Adverse childhood experiences and hormonal contraception: Interactive impact on sexual reward function
Source: PLoS One. 2023 Jan 17;18(1):e0279764. doi: 10.1371/journal.pone.0279764 (PMC9844925; doi:10.1371/journal.pone.0279764)
Supplement: S1 File — (DOCX) [file pone.0279764.s001.docx]

**Supplemental Materials**

**Key inclusion criteria:**

1. Aged 18-40 years of age
2. Cisgender females (assigned female at birth and reported identifying as female)
3. In generally good health per self-report. Those with a chronic medical condition were eligible as long as it was stable*
4. For women in the hormonal contraception group: they needed to have been on the same hormonal contraception for at least 3 months
5. For women in the non-hormonal contraception group: they needed to have been off hormonal contraception for at least 3 months

**Key exclusion criteria:**

1. Presence or history of severe or unstable physical, neurological or psychiatric condition (per self-report on health history survey)
2. Reported being pregnant or having a partner who was currently pregnant
3. For women in the non-hormonal contraception group: they reported having a menstrual cycle length that was less than 21 days or more than 35 days

*Participants were asked to rate their current health as either “poor,” “fair,” “good,” “very good,” or “excellent.” Individuals who rated their health as “fair” or “poor” were excluded. Similarly, if participants endorsed that they had any current medical conditions, they were subsequently asked the severity of these conditions and to what extent they were under control and affected their current life. Only participants who indicated disorders that were moderately severe or severe and not under control were excluded.

**Appropriateness of dichotomizing ACEs**

Following analysis of data using the dichotomization of individuals into low ACE (0-1 ACEs) and high ACE (2+ ACEs) groups, we plotted the relationship between number of prepubertal ACEs and expected valence ratings for erotic images in both the HC and No HC group to ensure that dichotomization was appropriate. As below, this revealed a non-linear relationship, suggesting that the dichotomization of 0-1 and 2+ resulted in more conservative estimates of the effects of ACEs compared to if they had been treated as a continuous variable.


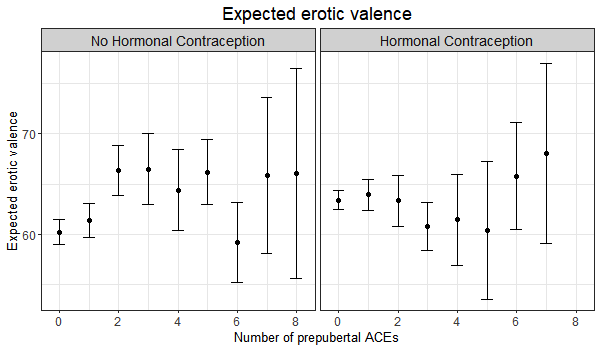


**Survey question on side effects for women currently taking hormonal contraception:**

Do you experience any of the following side effects from your hormonal contraception?

Physical side effects (e.g., nausea, sore breasts, weight gain)

Mood changes (e.g., feeling down or anxious)

Decreased libido/sexual desire

Other/none of the above

No side effects

**Survey questions on reason for discontinuation for former users of hormonal contraception:**

Why did you stop using this contraceptive method?

Side effects (e.g., depressed mood, weight gain, decreased libido)

Cost

Logistics (e.g., remembering to take pill)

No longer needed/wanted it (e.g., no risk of pregnancy, trying to get pregnant)

Other

**(If indicated side effects as reason, then asked following question):**

Did you stop taking hormonal contraceptives because of any of the side effects?

Physical side effects (e.g., nausea, sore breasts, weight gain)

Mood changes (e.g., feeling down or anxious)

Decreased libido/sexual desire

Other/none of the above

**Selection and validation of images used in reward task**

In order to determine appropriate images to be used in future tasks, 130 women ranging from 18-35 years old rated images. Women were recruited via internet listservs, word of mouth, flyers, and brochures. To be eligible, participants had to report: having regular menstrual cycles (ranging from 24-32 days) for the past 6 months, not taking any kind of hormonal treatment (including hormonal contraception) for the past 6 months, and being in generally good health (no severe or unstable physical, neurological, or psychiatric disorder). Half of the women (n=65) participated during the late follicular phase of their menstrual cycle (defined as 9-14 days after the first day of last menstruation), and the other half participated during the mid- to late luteal phase (defined as 19-23 days after first day of last menstruation). All menstrual phase determination was done by self-report.

Initially selected images were from either publicly available sources on the internet or affective image sets used in research (49–51). The images that were selected consisted of 40 erotic images (males and females in various states of nudity, either alone or engaged in sexual activity together), 40 non-erotic pleasant images (e.g., nature scenes, “cute” animals), 40 neutral images (e.g., random inanimate objects, such as furniture), and 40 unpleasant images (e.g., wounds, war, violence) for a total of 160 images.

Testing occurred remotely over the internet. Participants rated each image on a scale from 1 = *very unpleasant* to 9 = *very pleasant* (referred to as the valence of the image). Each image was presented for 10 seconds. Images were presented in blocks of 40 images. For all participants, the first block was the erotic images in order to prevent contamination effects from other image categories, particularly the unpleasant images. The images for the next 3 blocks were a mixed assortment of non-erotic pleasant, neutral, and unpleasant images.

When planning the present study, preliminary data from 34 participants was used to inform selection of 20 of the original 40 images in each category for use in the current reward task. In order to select images that most consistently received ratings appropriate for their category, the 20 images in each category with the lowest standard deviation were used. Among the 20 erotic images with the lowest standard deviations, there were two images with a mean valence below 4 so these were replaced with images with ratings above 4 with the next lowest standard deviations in an effort to avoid utilizing unpleasant erotic images. Average mean valence for chosen images in each category were: erotic = 5.44 (range 4.28-6.97); non-erotic pleasant = 7.09 (range 6.58-7.46); neutral = 3.84 (range 3.33-4.51). The resulting selected erotic images consisted of 5 images of shirtless males, 7 images of heterosexual couples engaging in intimacy who were clothed or partially clothed without visible genitalia or female breasts, and 6 images of nude heterosexual couples engaging in intimacy (with visible female breasts but without overtly visible genitalia).

**Selection of covariates**

Demographic variables that were associated with image ratings and differed according to HC and/or ACE group (*p* < 0.1) were subsequently incorporated into analyses as covariates. The image randomization group that participants were assigned to was also included in all adjusted models to control for any potential randomization effects.

**Table S1. Demographic of study completers stratified by prepubertal adverse childhood experiences (ACE) group.**

| **Demographics variable**  N (%) or mean (SD) | **Study completers**  (N = 1029) | **Low ACE**  (N = 743) | **High ACE**  (N = 286) | **p-value** |
| --- | --- | --- | --- | --- |
| Current HC use |  |  |  | 0.094 |
| HC use | 541 | 403 | 138 |  |
| No HC use | 488 | 340 | 148 |  |
| Age | 28.7 (5.2) | 28.5 (5.0) | 29.2 (5.7) | 0.063 |
| Race |  |  |  | 0.384 |
| Asian | 78 (7.6%) | 56 (7.5%) | 22 (7.7%) |  |
| Black or African American | 40 (3.9%) | 27 (3.6%) | 13 (4.5%) |  |
| White | 844 (82.0%) | 617 (83.0%) | 227 (79.4%) |  |
| Other/multiracial | 67 (6.5%) | 43 (5.8%) | 24 (8.4%) |  |
| Ethnicity |  |  |  | 0.001 |
| Hispanic or Latino | 74 (7.2%) | 60 (8.1%) | 14 (4.9%) |  |
| Non-Hispanic or Non-Latino | 938 (91.2%) | 677 (91.1%) | 261 (91.3%) |  |
| Other or multiple ethnicities | 17 (1.7%) | 6 (0.8%) | 11 (3.8%) |  |
| Sexual orientation |  |  |  | 0.002 |
| Heterosexual | 802 (77.9%) | 601 (80.9%) | 201 (70.3%) |  |
| Bisexual | 156 (15.2%) | 92 (12.4%) | 64 (22.4%) |  |
| Homosexual/gay/lesbian | 35 (3.4%) | 24 (3.2%) | 11 (3.8%) |  |
| Other | 36 (3.5%) | 26 (3.5%) | 10 (3.5%) |  |
| Relationship status |  |  |  | 0.122 |
| In a relationship | 684 (66.5%) | 483 (65.0%) | 201 (70.3%) |  |
| Single | 345 (33.5%) | 260 (35.0%) | 85 (29.7%) |  |
| Household income |  |  |  | 0.375 |
| Less than $25,000 | 76 (7.4%) | 54 (7.3%) | 22 (7.7%) |  |
| $25,000 - $75,000 | 410 (39.8%) | 295 (39.7%) | 115 (40.2%) |  |
| $75,000 - $200,000 | 461 (44.8%) | 328 (44.1%) | 133 (46.5%) |  |
| $200,000 or more | 82 (8.0%) | 66 (8.9%) | 16 (5.6%) |  |
| Highest education level |  |  |  | 0.001 |
| High school diploma or less | 172 (16.7%) | 107 (14.4%) | 65 (22.7%) |  |
| College degree | 499 (48.5%) | 356 (47.9%) | 143 (50.0%) |  |
| Master’s/professional degree | 358 (34.8%) | 280 (37.7%) | 78 (27.3%) |  |
| Prepubertal ACEs | 1.2 (1.6) | 0.3 (0.5) | 3.3 (1.4) | <0.001 |
| HC Type* |  |  |  | 0.966 |
| Hormonal IUD | 227 (45.6%)* | 166 (45.2%)* | 61 (46.6%)* |  |
| Progestin + Estrogen | 223 (44.8%)* | 165 (45.0%)* | 58 (44.3%)* |  |
| Progestin only | 48 (9.6%)* | 36 (9.8%)* | 12 (9.2%)* |  |

*Continuous variables are summarized with means (standard deviations), with differences among groups tested using two-sample t-tests. Categorical variables are summarized with frequencies (percentages), with differences among groups tested using Fisher’s exact tests. Low ACE = 0 to 1 ACEs. High ACE = 2+ ACEs. *HC Type is relevant only to current HC users who provided adequate information on their HC for categorization (n = 498). Of the 498, 367 met criteria as Low ACE and 131 met criteria as High ACE.*

**Selection of covariates**

Demographic variables that were associated with image ratings and differed according to HC and/or ACE group (*p* < 0.1) were subsequently incorporated into analyses as covariates. The image randomization group that participants were assigned to was also included in all adjusted models to control for any potential randomization effects

**Table S2. Type III p values for each demographic variable and their association with expected and experienced valence ratings (overall and by image type).**

|  | **Overall** | | **Neutral** | | **Pleasant** | | **Erotic** | |
| --- | --- | --- | --- | --- | --- | --- | --- | --- |
| **Demographic variable or potential covariate of interest** | **Expected valence: p value** | **Experienced valence: p value** | **Expected valence: p value** | **Experienced valence: p value** | **Expected valence: p value** | **Experienced valence: p value** | **Expected valence: p value** | **Experienced valence: p value** |
| Age | 0.900 | 0.516**^a^** | 0.083**^a^** | 0.573 | **<0.001^a^** | 0.075**^a^** | 0.073^a^ | 0.098**^a^** |
| Race | 0.067 | 0.118 | 0.702 | 0.485 | 0.148 | 0.171 | 0.092**^a^** | 0.058**^a^** |
| Ethnicity | 0.227 | 0.162 | 0.471 | 0.288 | **0.043^a^** | 0.177 | 0.939 | 0.113 |
| Sexual orientation | **0.006^a^** | **<0.001^a^** | 0.973 | **0.019^a^** | 0.178 | 0.734 | **<0.001^a^** | **<0.001^a^** |
| First language | 0.755 | **0.035^a^** | 0.980 | 0.114 | 0.693 | 0.261 | 0.925 | 0.268 |
| Relationship status | 0.475 | 0.332 | 0.743 | 0.725 | 0.774 | 0.342 | 0.289 | 0.313 |
| Household income | 0.625 | 0.577 | 0.291 | 0.516 | 0.345 | 0.133 | **0.031^a^** | 0.182 |
| Education | 0.744 | 0.173 | 0.643 | 0.379 | 0.343 | **0.010^a^** | 0.670 | 0.292 |
| Randomization group | 0.174 | 0.612 | 0.302 | **0.011^a^** | **<0.001^a^** | 0.458 | 0.893 | 0.945 |

**^a^** Meets criteria for covariate consideration with p < 0.1

**Table S3. Demographic variables meeting criteria for selection as covariates due to both an association with valence ratings (Table S2) and with hormonal contraception (HC) use (Table 1) and/or prepubertal adverse childhood experiences (ACE) group (Table S1).**

|  | **Associated with valence and differs by HC group and/or ACE group?** |
| --- | --- |
| Age | X |
| Race | X |
| Ethnicity* | X |
| Sexual orientation | X |
| First language |  |
| Relationship status |  |
| Household income |  |
| Education | X |
| Randomization group** | X |

**Ethnicity was not included due to race and ethnicity being highly associated (p < 0.001).*

***Although image randomization group did not differ by HC use (p = 0.878) or ACE group (p = 0.137), it was nevertheless included as a covariate in all analyses to account for potential order effects of image presentation.*

**Table S4. Interaction effect of hormonal contraception (HC) use and prepubertal adverse childhood experiences (ACE) group on expected and experienced valence of neutral, pleasant, and erotic image ratings in full models adjusting for covariates.**

| **Predictor** | **Estimate (95% CI)** | **p value** |
| --- | --- | --- |
| **Expected: neutral** |  |  |
| High prepubertal ACE (ref: low) | -0.30 (-2.34, 1.75) | 0.777 |
| Hormonal birth control (ref: no HC) | -1.12 (-2.64, 0.40) | 0.149 |
| Asian race (ref: White) | 1.46 (-0.98, 3.90) | 0.240 |
| Other/multiracial race (ref: White) | 0.84 (-1.77, 3.46) | 0.527 |
| Black/African American race (ref: White) | -0.82 (-4.14, 2.50) | 0.628 |
| Bisexual orientation (ref: Heterosexual) | 0.15 (-1.67, 1.97) | 0.873 |
| Other sexual orientation (ref: Heterosexual) | 0.84 (-2.66, 4.34) | 0.639 |
| Homosexual/gay/lesbian orientation (ref: Heterosexual) | 0.12 (-3.44, 3.68) | 0.947 |
| College degree (ref: HS degree or less) | -0.67 (-2.52, 1.18) | 0.479 |
| Masters/professional degree (ref: HS degree or less) | -0.46 (-2.56, 1.65) | 0.671 |
| Age | 0.12 (-0.02, 0.26) | 0.086 |
| Randomization group 2 (ref: 1) | -0.21 (-2.03, 1.61) | 0.822 |
| Randomization group 3 (ref: 1) | -0.88 (-2.70, 0.95) | 0.346 |
| Randomization group 4 (ref: 1) | -1.60 (-3.42, 0.23) | 0.086 |
| ACE * HC interaction | 0.37 (-2.48, 3.22) | 0.802 |
| **Expected: pleasant** |  |  |
| High prepubertal ACE (ref: low) | 1.46 (-1.37, 4.28) | 0.312 |
| Hormonal birth control (ref: no HC) | 0.02 (-2.08, 2.12) | 0.983 |
| Asian race (ref: White) | -2.27 (-5.64, 1.09) | 0.185 |
| Other/multiracial race (ref: White) | -1.61 (-5.22, 2.00) | 0.381 |
| Black/African American race (ref: White) | -3.44 (-8.02, 1.14) | 0.141 |
| Bisexual orientation (ref: Heterosexual) | 0.11 (-2.40, 2.63) | 0.928 |
| Other sexual orientation (ref: Heterosexual) | 3.40 (-1.43, 8.23) | 0.167 |
| Homosexual/gay/lesbian orientation (ref: Heterosexual) | 3.65 (-1.26, 8.55) | 0.145 |
| College degree (ref: HS degree or less) | 2.61 (0.06, 5.16) | 0.045 |
| Masters/professional degree (ref: HS degree or less) | 3.24 (0.33, 6.14) | 0.029 |
| Age | -0.38 (-0.57, -0.18) | <0.001 |
| Randomization group 2 (ref: 1) | 0.49 (-2.03, 3.00) | 0.705 |
| Randomization group 3 (ref: 1) | 5.22 (2.70, 7.74) | <0.001 |
| Randomization group 4 (ref: 1) | 1.31 (-1.20, 3.83) | 0.306 |
| ACE * HC interaction | -0.53 (-4.46, 3.41) | 0.792 |
| **Expected: erotic** |  |  |
| High prepubertal ACE (ref: low) | 4.00 (0.63, 7.36) | 0.020 |
| Hormonal birth control (ref: no HC) | 2.28 (-0.22, 4.79) | 0.074 |
| Asian race (ref: White) | -2.47 (-6.48, 1.54) | 0.226 |
| Other/multiracial race (ref: White) | -1.83 (-6.13, 2.47) | 0.404 |
| Black/African American race (ref: White) | -5.06 (-10.52, 0.40) | 0.069 |
| Bisexual orientation (ref: Heterosexual) | 3.58 (0.59, 6.57) | 0.019 |
| Other sexual orientation (ref: Heterosexual) | -13.90 (-19.66, -8.15) | <0.001 |
| Homosexual/gay/lesbian orientation (ref: Heterosexual) | -13.11 (-18.96, -7.26) | <0.001 |
| College degree (ref: HS degree or less) | -0.38 (-3.43, 2.66) | 0.805 |
| Masters/professional degree (ref: HS degree or less) | -1.90 (-5.37, 1.56) | 0.281 |
| Age | 0.25 (0.02, 0.48) | 0.035 |
| Randomization group 2 (ref: 1) | -0.06 (-3.06, 2.93) | 0.967 |
| Randomization group 3 (ref: 1) | -1.32 (-4.32, 1.68) | 0.388 |
| Randomization group 4 (ref: 1) | 1.14 (-1.86, 4.14) | 0.455 |
| ACE * HC interaction | -5.27 (-9.95, -0.58) | 0.028 |
| **Experienced: neutral** |  |  |
| High prepubertal ACE (ref: low) | 0.09 (-1.68, 1.85) | 0.924 |
| Hormonal birth control (ref: no HC) | -0.52 (-1.83, 0.80) | 0.441 |
| Asian race (ref: White) | 1.69 (-0.42, 3.79) | 0.116 |
| Other/multiracial race (ref: White) | 0.45 (-1.81, 2.71) | 0.698 |
| Black/African American race (ref: White) | 0.28 (-2.59, 3.15) | 0.849 |
| Bisexual orientation (ref: Heterosexual) | 1.09 (-0.48, 2.67) | 0.172 |
| Other sexual orientation (ref: Heterosexual) | 4.58 (1.55, 7.60) | 0.003 |
| Homosexual/gay/lesbian orientation (ref: Heterosexual) | 1.95 (-1.12, 5.03) | 0.212 |
| College degree (ref: HS degree or less) | 0.92 (-0.68, 2.51) | 0.261 |
| Masters/professional degree (ref: HS degree or less) | 1.33 (-0.49, 3.15) | 0.151 |
| Age | 0.00 (-0.12, 0.12) | 0.984 |
| Randomization group 2 (ref: 1) | 1.48 (-0.09, 3.05) | 0.065 |
| Randomization group 3 (ref: 1) | 0.82 (-0.76, 2.39) | 0.310 |
| Randomization group 4 (ref: 1) | -1.19 (-2.76, 0.39) | 0.140 |
| ACE * HC interaction | 0.46 (-2.00, 2.92) | 0.715 |
| **Experienced: pleasant** |  |  |
| High prepubertal ACE (ref: low) | -0.20 (-2.03, 1.63) | 0.832 |
| Hormonal birth control (ref: no HC) | 0.26 (-1.10, 1.62) | 0.710 |
| Asian race (ref: White) | -0.25 (-2.43, 1.93) | 0.824 |
| Other/multiracial race (ref: White) | -1.48 (-3.81, 0.86) | 0.215 |
| Black/African American race (ref: White) | -2.63 (-5.60, 0.34) | 0.082 |
| Bisexual orientation (ref: Heterosexual) | -0.09 (-1.71, 1.54) | 0.916 |
| Other sexual orientation (ref: Heterosexual) | 0.28 (-2.85, 3.41) | 0.862 |
| Homosexual/gay/lesbian orientation (ref: Heterosexual) | 1.55 (-1.63, 4.73) | 0.338 |
| College degree (ref: HS degree or less) | -2.24 (-3.89, -0.58) | 0.008 |
| Masters/professional degree (ref: HS degree or less) | -1.89 (-3.78, -0.01) | 0.049 |
| Age | -0.06 (-0.18, 0.07) | 0.373 |
| Randomization group 2 (ref: 1) | 0.86 (-0.77, 2.49) | 0.300 |
| Randomization group 3 (ref: 1) | 1.34 (-0.29, 2.97) | 0.107 |
| Randomization group 4 (ref: 1) | 1.01 (-0.62, 2.64) | 0.225 |
| ACE * HC interaction | -0.48 (-3.03, 2.07) | 0.713 |
| **Experienced: erotic** |  |  |
| High prepubertal ACE (ref: low) | 2.84 (-0.14, 5.82) | 0.062 |
| Hormonal birth control (ref: no HC) | 2.63 (0.41, 4.85) | 0.020 |
| Asian race (ref: White) | -2.02 (-5.57, 1.53) | 0.264 |
| Other/multiracial race (ref: White) | 1.86 (-1.95, 5.68) | 0.337 |
| Black/African American race (ref: White) | -7.26 (-12.10, -2.42) | 0.003 |
| Bisexual orientation (ref: Heterosexual) | 1.08 (-1.57, 3.73) | 0.425 |
| Other sexual orientation (ref: Heterosexual) | -14.98 (-20.08, -9.88) | <0.001 |
| Homosexual/gay/lesbian orientation (ref: Heterosexual) | -18.99 (-24.18, -13.81) | <0.001 |
| College degree (ref: HS degree or less) | -1.97 (-4.66, 0.73) | 0.153 |
| Masters/professional degree (ref: HS degree or less) | -3.18 (-6.25, -0.11) | 0.042 |
| Age | 0.24 (0.03, 0.44) | 0.022 |
| Randomization group 2 (ref: 1) | -1.11 (-3.76, 1.55) | 0.413 |
| Randomization group 3 (ref: 1) | -1.22 (-3.88, 1.44) | 0.368 |
| Randomization group 4 (ref: 1) | 0.08 (-2.58, 2.74) | 0.954 |
| ACE * HC interaction | -4.76 (-8.91, -0.60) | 0.025 |

**Table S5. Among current hormonal contraception (HC) users, self-reported HC-induced decreases in sexual desire interacted with prepubertal adverse childhood experiences (ACEs) to predict expected but not experienced valence ratings for erotic images.** **Among former hormonal contraception (HC) users, self-reported discontinuation of HC due to decreases in sexual desire and prepubertal adverse childhood experiences (ACEs) did not interact to predict expected or experienced valence ratings for erotic images.**

|  |  |  | **Low ACE** | | | **High ACE** | | |
| --- | --- | --- | --- | --- | --- | --- | --- | --- |
|  | **Interaction**  **p-value** | **χ^2^**  $\boldsymbol{(df=1)}$ | **Decreased sexual desire estimate (95% CI)** | **Z** | **p-value** | **Decreased sexual desire estimate (95% CI)** | **Z** | **p-value** |
| **Current HC users** |  |  |  |  |  |  |  |  |
| Expected | **0.008** | 7.11 | -1.50 (-5.23, 2.22) | -0.79 | 0.429 | **-10.81 (-16.53, -5.08)** | **-3.70** | **<0.001** |
| Experienced | 0.195 | 1.67 | -1.29 (-4.58, 2.01) | -0.77 | 0.443 | **-5.28 (-10.35, -0.21)** | **-2.04** | **0.041** |
| **Former HC users** |  |  |  |  |  |  |  |  |
| Expected | 0.761 | 0.09 | 1.24 (-4.17, 6.65) | 0.45 | 0.653 | 2.65 (-4.64, 9.94) | 0.71 | 0.476 |
| Experienced | 0.335 | 0.93 | 1.43 (-3.48, 6.33) | 0.57 | 0.569 | 5.49 (-1.12, 12.11) | 1.63 | 0.104 |

*For expected, but not experienced valence ratings of erotic images in Current HC users, there was a significant interaction such that those in the high prepubertal ACE group, who reported decreased sexual desire, had substantially lower anticipated ratings of erotic images compared to women without decreased sexual desire. Interactions were not significant in Former HC users. All models controlled for randomization group, race, age, education, and sexual orientation. Low ACE = 0 to 1 ACEs. High ACE = 2+ ACEs.*

*.*

**Table S6. Among current hormonal contraception (HC) users, interaction of prepubertal adverse childhood experiences (ACE) group and self-reported HC-induced decreases in sexual desire (yes vs. no) on expected and experienced valence of erotic image ratings in full models adjusting for covariates.**

| **Outcome: expected erotic valence** | **Estimate (95% CI)** | **p value** |
| --- | --- | --- |
| High prepubertal ACE (ref: low) | 1.64 (-2.07, 5.34) | 0.386 |
| Experiences decrease in sexual desire on HC (ref: no decrease) | -1.50 (-5.24, 2.23) | 0.429 |
| Asian race (ref: White) | -5.45 (-11.24, 0.34) | 0.065 |
| Other/multiracial race (ref: White) | -1.08 (-6.72, 4.56) | 0.708 |
| Black/African American race (ref: White) | -6.32 (-14.85, 2.22) | 0.146 |
| Bisexual orientation (ref: Heterosexual) | 4.53 (0.67, 8.39) | 0.022 |
| Other sexual orientation (ref: Heterosexual) | -10.06 (-17.33, -2.80) | 0.007 |
| Homosexual/gay/lesbian orientation (ref: Heterosexual) | -13.27 (-22.84, -3.69) | 0.007 |
| College degree (ref: HS degree or less) | 4.76 (0.34, 9.18) | 0.035 |
| Masters/professional degree (ref: HS degree or less) | 0.22 (-4.71, 5.15) | 0.93 |
| Age | 0.00 (-0.31, 0.32) | 0.981 |
| Randomization group 2 (ref: 1) | -0.79 (-4.59, 3.01) | 0.683 |
| Randomization group 3 (ref: 1) | -1.00 (-4.90, 2.90) | 0.615 |
| Randomization group 4 (ref: 1) | -0.01 (-3.86, 3.83) | 0.995 |
| ACE * Decrease in sexual desire interaction | -9.30 (-16.15, -2.45) | 0.008 |
| **Outcome: experienced erotic valence** | **Estimate (95% CI)** | **p value** |
| High prepubertal ACE (ref: low) | -0.78 (-4.05, 2.50) | 0.642 |
| Experiences decrease in sexual desire on HC (ref: no decrease) | -1.29 (-4.59, 2.01) | 0.444 |
| Asian race (ref: White) | -6.64 (-11.77, -1.52) | 0.011 |
| Other/multiracial race (ref: White) | 3.49 (-1.50, 8.48) | 0.170 |
| Black/African American race (ref: White) | -8.97 (-16.52, -1.42) | 0.020 |
| Bisexual orientation (ref: Heterosexual) | 1.86 (-1.56, 5.27) | 0.286 |
| Other sexual orientation (ref: Heterosexual) | -10.96 (-17.38, -4.53) | <0.001 |
| Homosexual/gay/lesbian orientation (ref: Heterosexual) | -18.77 (-27.24, -10.30) | <0.001 |
| College degree (ref: HS degree or less) | 0.64 (-3.27, 4.55) | 0.748 |
| Masters/professional degree (ref: HS degree or less) | -3.32 (-7.68, 1.04) | 0.135 |
| Age | 0.18 (-0.10, 0.46) | 0.216 |
| Randomization group 2 (ref: 1) | -2.67 (-6.03, 0.69) | 0.119 |
| Randomization group 3 (ref: 1) | -0.97 (-4.42, 2.48) | 0.581 |
| Randomization group 4 (ref: 1) | -0.93 (-4.33, 2.47) | 0.590 |
| ACE * Decrease in sexual desire interaction | -3.99 (-10.05, 2.07) | 0.196 |

**Table S7. Among former hormonal contraception (HC) users, interaction of prepubertal adverse childhood experiences (ACE) group and self-reported HC-induced decreased sexual desire (yes vs. no) on expected and experienced valence of erotic image ratings in full models adjusting for covariates.**

| **Outcome: expected erotic valence** | **Estimate (95% CI)** | **p value** |
| --- | --- | --- |
| High prepubertal ACE (ref: low) | 2.91 (-1.90, 7.72) | 0.235 |
| Stopped HC due to decrease in sexual desire (ref: did not) | 1.24 (-4.19, 6.67) | 0.653 |
| Asian race (ref: White) | -6.46 (-15.38, 2.45) | 0.155 |
| Other/multiracial race (ref: White) | -3.06 (-10.23, 4.11) | 0.402 |
| Black/African American race (ref: White) | -2.81 (-11.45, 5.83) | 0.523 |
| Bisexual orientation (ref: Heterosexual) | 0.18 (-5.19, 5.55) | 0.948 |
| Other sexual orientation (ref: Heterosexual) | -18.03 (-29.00, -7.06) | 0.001 |
| Homosexual/gay/lesbian orientation (ref: Heterosexual) | -11.62 (-26.36, 3.12) | 0.122 |
| College degree (ref: HS degree or less) | -3.59 (-9.32, 2.14) | 0.218 |
| Masters/professional degree (ref: HS degree or less) | -0.38 (-6.61, 5.85) | 0.904 |
| Age | 0.01 (-0.42, 0.43) | 0.976 |
| Randomization group 2 (ref: 1) | -4.42 (-9.97, 1.12) | 0.117 |
| Randomization group 3 (ref: 1) | -5.27 (-10.83, 0.29) | 0.063 |
| Randomization group 4 (ref: 1) | -2.56 (-8.12, 3.00) | 0.366 |
| ACE * Discontinuation due to decrease in sexual desire interaction | 1.41 (-7.73, 10.56) | 0.761 |
| **Outcome: experienced erotic valence** | **Estimate (95% CI)** | **p value** |
| High prepubertal ACE (ref: low) | 0.64 (-3.72, 5.01) | 0.772 |
| Stopped HC due to decrease in sexual desire (ref: did not) | 1.43 (-3.50, 6.35) | 0.570 |
| Asian race (ref: White) | -1.85 (-9.94, 6.24) | 0.653 |
| Other/multiracial race (ref: White) | 1.85 (-4.66, 8.35) | 0.576 |
| Black/African American race (ref: White) | -5.39 (-13.23, 2.45) | 0.177 |
| Bisexual orientation (ref: Heterosexual) | -0.03 (-4.90, 4.85) | 0.992 |
| Other sexual orientation (ref: Heterosexual) | -21.35 (-31.31, -11.39) | <0.001 |
| Homosexual/gay/lesbian orientation (ref: Heterosexual) | -22.78 (-36.15, -9.41) | <0.001 |
| College degree (ref: HS degree or less) | -1.68 (-6.88, 3.52) | 0.524 |
| Masters/professional degree (ref: HS degree or less) | 0.52 (-5.14, 6.17) | 0.857 |
| Age | -0.08 (-0.47, 0.31) | 0.685 |
| Randomization group 2 (ref: 1) | -4.76 (-9.79, 0.27) | 0.064 |
| Randomization group 3 (ref: 1) | -6.56 (-11.61, -1.52) | 0.011 |
| Randomization group 4 (ref: 1) | -5.30 (-10.34, -0.25) | 0.040 |
| ACE * Discontinuation due to decrease in sexual desire interaction | 4.07 (-4.23, 12.36) | 0.335 |

**Table S8.** **Hormonal contraception (HC) type in current HC users in the high adverse child experience (ACE) group summarized overall and by whether they experienced decreased sexual desire as a side effect.**

|  | **Overall**  (N = 138) | **No current decrease in sexual desire**  (N = 95) | **Current decrease in sexual desire** (N = 43) | **p value** |
| --- | --- | --- | --- | --- |
| HC type |  |  |  | 0.202 |
| Hormonal IUD | 61 (46.6%) | 47 (51.6%) | 14 (35.0%) |  |
| Progestin. + estrogen | 58 (44.3%) | 36 (39.6%) | 22 (55.0%) |  |
| Progestin only | 12 (9.2%) | 8 (8.8%) | 4 (10.0%) |  |

*Categorical variables are summarized with frequencies (percentages), with differences among groups tested using Fisher’s exact tests. Low ACE = 0 to 1 ACEs. High ACE = 2+ ACEs.*

**Table S9.** **Demographics of the high prepubertal adverse childhood experience (ACE) group currently taking hormonal contraception (HC) stratified by reported decreased sexual desire as a side effect.**

| **Demographics (N [%] or mean [SD])** | **High ACE females currently on HC** (N = 138) | **No current decrease in sexual desire**  (N = 95) | **Current decrease in sexual desire**  (N = 43) | **p value** |
| --- | --- | --- | --- | --- |
| Age | 28.4 (5.2) | 28.2 (5.1) | 28.8 (5.4) | 0.525 |
| Race |  |  |  | 0.536 |
| Asian | 11 (8.0%) | 9 (9.5%) | 2 (4.7%) |  |
| Black/African American | 4 (2.9%) | 3 (3.2%) | 1 (2.3%) |  |
| White | 111 (80.4%) | 73 (76.8%) | 38 (88.4%) |  |
| Other/multiracial | 12 (8.7%) | 10 (10.5%) | 2 (4.7%) |  |
| Ethnicity |  |  |  | 0.697 |
| Not Hispanic/Latino | 130 (94.2%) | 88 (92.6%) | 42 (97.7%) |  |
| Hispanic/Latino | 5 (3.6%) | 4 (4.2%) | 1 (2.3%) |  |
| Other/multiple ethnicities | 3 (2.2%) | 3 (3.2%) | 0 (0.0%) |  |
| Sexual orientation |  |  |  | 0.979 |
| Heterosexual | 99 (71.7%) | 67 (70.5%) | 32 (74.4%) |  |
| Bisexual | 28 (20.3%) | 20 (21.1%) | 8 (18.6%) |  |
| Homosexual/gay/lesbian | 4 (2.9%) | 3 (3.2%) | 1 (2.3%) |  |
| Other | 7 (5.1%) | 5 (5.3%) | 2 (4.7%) |  |
| Relationship status |  |  |  | 1 |
| In a relationship | 104 (75.4%) | 71 (74.7%) | 33 (76.7%) |  |
| Single | 34 (24.6%) | 24 (25.3%) | 10 (23.3%) |  |
| Household income |  |  |  | 0.675 |
| Less than $25,000 | 7 (5.1%) | 5 (5.3%) | 2 (4.7%) |  |
| $25,000-$75,000 | 54 (39.1%) | 38 (40.0%) | 16 (37.2%) |  |
| $75,000-$200,000 | 69 (50.0%) | 45 (47.4%) | 24 (55.8%) |  |
| $200,000 or more | 8 (5.8%) | 7 (7.4%) | 1 (2.3%) |  |
| Highest level of education |  |  |  | 0.934 |
| High school diploma or less | 24 (17.4%) | 16 (16.8%) | 8 (18.6%) |  |
| College degree | 76 (55.1%) | 52 (54.7%) | 24 (55.8%) |  |
| Master's/professional degree | 38 (27.5%) | 27 (28.4%) | 11 (25.6%) |  |

*Continuous variables are summarized with means (standard deviations), with differences among groups tested using two-sample t-tests. Categorical variables are summarized with frequencies (percentages), with differences among groups tested using Fisher’s exact tests. High ACE = 2+ ACEs.*

**Table S10. Hormonal contraception (HC) use does not interact with prepubertal sexual abuse to predict expected and experienced valence of erotic, neutral, or pleasant images.**

|  |  |  | **No prepubertal sexual abuse** | | | **Prepubertal sexual abuse** | | |
| --- | --- | --- | --- | --- | --- | --- | --- | --- |
| **Condition Type** | **Interaction**  **p-value** | **Interaction** $\boldsymbol{\chi}^{\boldsymbol{2}}\boldsymbol{, df}$ | **HC estimate (95% CI)** | **Z** | **p-value** | **HC estimate (95% CI)** | **Z** | **p-value** |
| **Expected** |  |  |  |  |  |  |  |  |
| Neutral | 0.692 | 0.15, 1 | -1.05 (-2.40, 0.30) | -1.53 | 0.126 | -0.05 (-4.85, 4.75) | -0.02 | 0.985 |
| Pleasant | 0.650 | 0.21, 1 | 0.03 (-1.84, 1.89) | 0.03 | 0.977 | -1.57 (-8.19, 5.06) | -0.46 | 0.643 |
| Erotic | 0.524 | 0.41, 1 | 0.95 (-1.28, 3.17) | 0.83 | 0.405 | -1.73 (-9.64, 6.19) | -0.43 | 0.669 |
| **Experienced** |  |  |  |  |  |  |  |  |
| Neutral | 0.732 | 0.12, 1 | -0.41 (-1.58, 0.75) | -0.69 | 0.488 | 0.34 (-3.81, 4.49) | 0.16 | 0.872 |
| Pleasant | 0.699 | 0.15, 1 | 0.14 (-1.06, 1.35) | 0.23 | 0.817 | -0.74 (-5.02, 3.55) | -0.34 | 0.737 |
| Erotic | 0.793 | 0.07, 1 | 1.15 (-0.82, 3.13) | 1.15 | 0.252 | 2.13 (-4.88, 9.14) | 0.60 | 0.552 |

*The interaction effect was tested separately for each image type within each condition type, and all models controlled for randomization group, race, age, education, and sexual orientation. \*

**Table S11. Among current hormonal contraception (HC) users and former HC users, self-reported HC-induced decreases in sexual desire did not interact with prepubertal sexual abuse to predict expected or experienced valence ratings for erotic images.**

|  |  |  | **No prepubertal sexual abuse** | | | **Prepubertal sexual abuse** | | |
| --- | --- | --- | --- | --- | --- | --- | --- | --- |
|  | **Interaction**  **p-value** | $\boldsymbol{\chi}^{\boldsymbol{2}}$  $\boldsymbol{(df=1)}$ | **Decreased sexual desire estimate (95% CI)** | **Z** | **p-value** | **Decreased sexual desire estimate (95% CI)** | **Z** | **p-value** |
| **Current HC users** |  |  |  |  |  |  |  |  |
| Expected | 0.950 | <0.01 | **-4.40 (-7.61, -1.19)** | **-2.68** | **0.007** | -3.95 (-17.58, 9.68) | -0.57 | 0.570 |
| Experienced | 0.996 | <0.01 | -2.66 (-5.49, 0.17) | -1.85 | 0.066 | -2.69 (-14.71, 9.32) | -0.44 | 0.661 |
| **Former HC users** |  |  |  |  |  |  |  |  |
| Expected | 0.681 | 0.17 | 1.52 (-3.20, 6.24) | 0.63 | 0.527 | 4.16 (-7.48, 15.80) | 0.70 | 0.484 |
| Experienced | 0.199 | 1.65 | 2.08 (-2.18, 6.33) | 0.96 | 0.339 | 9.51 (-0.98, 20.01) | 1.78 | 0.076 |

*All models controlled for randomization group, race, age, education, and sexual orientation.*

**Table S12. Lack of association between menstrual cycle phase (as determined by days since last reported menstrual period) and expected and experienced valence ratings of erotic images in women not taking hormonal contraception.**

| **Model (in N = 466 participants)** | **Unadjusted estimate (95% CI)** | **Pairwise p value** | **Overall**  **p value** | **Adjusted estimate (95% CI)** | **Pairwise p value** | **Overall p value** |
| --- | --- | --- | --- | --- | --- | --- |
| Expected erotic valence |  |  | 0.703 |  |  | 0.531 |
| Late follicular (ref: early follicular) | 0.18 (-4.15, 4.51) | 0.936 |  | 0.63 (-3.59, 4.85) | 0.770 |  |
| Luteal (ref: early follicular) | 1.62 (-2.74, 5.97) | 0.466 |  | 2.27 (-1.95, 6.49) | 0.291 |  |
| Experienced erotic valence |  |  | 0.961 |  |  | 0.968 |
| Late follicular (ref: early follicular) | -0.52 (-4.45, 3.41) | 0.796 |  | -0.05 (-3.81, 3.71) | 0.979 |  |
| Luteal (ref: early follicular) | -0.12 (-4.07, 3.84) | 0.953 |  | 0.37 (-3.40, 4.13) | 0.848 |  |

*Analysis consists of 466/488 participants who reported 35 days or less since last menstrual period. Early follicular phase = 0-7 days since last menstrual period; late follicular phase = 8-18 days since last menstrual period; luteal phase 19-35 days since last menstrual period. Adjusted for randomization group, race, age, education, and sexual orientation.*

References:

Kurdi, B., Lozano, S., Banaji, M.R., 2017. Introducing the Open Affective Standardized Image Set (OASIS). Behav Res 49, 457–470. <https://doi.org/10.3758/s13428-016-0715-3>

Lang, P., Bradley, M., Cuthbert, B., n.d. International affective picture system (IAPS): Affective ratings of pictures and instruction manual (Technical Report No. A-6). 2005. Gainesville, FL: The Center for Research in Psychophysiology, University of Florida.

Marchewka, A., Żurawski, Ł., Jednoróg, K., Grabowska, A., 2014. The Nencki Affective Picture System (NAPS): Introduction to a novel, standardized, wide-range, high-quality, realistic picture database. Behav Res 46, 596–610. <https://doi.org/10.3758/s13428-013-0379-1>
